# Estradiol and Raloxifene Protect Ovariectomized Mice from Acute Kidney Injury via G Protein-Coupled Estrogen Receptor-Mediated Nuclear Factor Erythroid 2-Related Factor 2/Heme Oxygenase-1 Activation

**DOI:** 10.3390/ijms27073070

**Published:** 2026-03-27

**Authors:** Yichuan Wang, Yanbo Song, Jingyu Dai, Xinxin Zhang, Lina Zhao, Yihua Mao, Maochao Ding

**Affiliations:** 1 Department of Anesthesiology, The First Affiliated Hospital of Wenzhou Medical University, Wenzhou 325000, China; wangyichuan008@163.com; 2 The First Clinical Medical College, Wenzhou Medical University, Wenzhou 325015, China; 3 Department of Anatomy, School of Basic Medical Sciences, Wenzhou Medical University, Wenzhou 325015, China

**Keywords:** acute kidney injury, G protein-coupled estrogen receptor, estradiol, raloxifene, heme oxygenase 1

## Abstract

Renal ischemia–reperfusion injury (IRI) is a major cause of acute kidney injury. Estradiol (E2) and the selective estrogen receptor modulator raloxifene (RAL) reduce organ dysfunction, potentially via heme oxygenase-1 (HO-1)–mediated antioxidant and anti-inflammatory effects. This study examined whether E2 and RAL protect against IRI through G protein-coupled estrogen receptor (GPER)–dependent activation of the nuclear factor erythroid 2-related factor 2 (Nrf2)/HO-1 pathway in ovariectomized (OVX) mice; OVX IRI mice were pretreated for four weeks with E2, RAL, RAL + ML385 (Nrf2 inhibitor), or RAL + G15 (GPER antagonist). Renal histology, inflammatory and oxidative markers, and nuclear Nrf2 levels were assessed; OVX IRI increased interleukin-1β (IL-1β), interleukin-6 (IL-6), tumor necrosis factor-alpha (TNF-α), and malondialdehyde (MDA) and decreased superoxide dismutase (SOD), catalase (CAT), and glutathione (GSH); nuclear Nrf2 was low in sham and OVX IRI groups. E2 and RAL improved renal function and histology, reduced inflammation and oxidative stress, restored GPER expression, increased nuclear Nrf2, and upregulated HO-1 and NAD(P)H:quinone oxidoreductase 1 (NQO1). Co-treatment with ML385 or G15 reversed RAL’s benefits, reduced nuclear Nrf2, and worsened injury; E2 and RAL exert renoprotective effects against OVX-related renal IRI in a manner consistent with GPER-dependent Nrf2 nuclear translocation, which suggests involvement of the downstream antioxidant gene activation pathway.

## 1. Introduction

Renal ischemia–reperfusion injury (IRI) is one of the main mechanisms leading to acute kidney injury (AKI). Ischemia activates renal endothelial cells and macrophages. These cells release pro-inflammatory cytokines such as tumor necrosis factor-alpha (TNF-α) and interleukin-6 (IL-6). The cytokines recruit neutrophils to infiltrate the renal parenchyma, causing damage. Ischemia impairs the normal function of the mitochondrial electron transport chain. Reperfusion leads to a sudden surge in oxygen supply, triggering an explosive production of reactive oxygen species (ROS). ROS directly attack cell membrane lipids and cellular DNA. When ROS production exceeds the scavenging capacity of endogenous antioxidant systems like superoxide dismutase (SOD), oxidative stress occurs. Oxidative stress induces lipid peroxidation and protein oxidative modification. It further exacerbates the injury and apoptosis of renal tubular epithelial cells. All these processes together drive the progression of AKI [1,2,3,4,5].

Raloxifene (RAL) is a widely used selective estrogen receptor modulator (SERM). It exhibits tissue-selective agonistic and antagonistic effects on estrogen receptors (ERs). In bone tissue, RAL acts as an ER agonist, making it effective against postmenopausal osteoporosis. In breast and uterine tissues, it functions as an ER antagonist, providing protection against breast cancer [6]. Additionally, RAL demonstrates systemic benefits, such as antioxidant and anti-inflammatory activities [7].

Estradiol (E2) primarily exerts its effects through classical estrogen receptors ERα and ERβ. However, a third receptor pathway exists via the G protein-coupled estrogen receptor (GPER). This pathway mediates many rapid non-genomic actions of E2. GPER is widely expressed in cardiovascular tissues, renal tissues, cerebral cortex, and reproductive organs [8]. GPER exerts functional roles in immune cells by inducing the expression of anti-inflammatory cytokine IL-10 and expanding regulatory T cell populations. In animal models, GPER deficiency promotes a pro-inflammatory state [9]. Studies identify GPER as a novel regulator of blood pressure and sodium homeostasis, where its activation significantly reduces blood pressure in male rats and induces vasodilation across multiple vascular beds, including coronary, carotid, aortic, and mesenteric arteries [10].

Furthermore, GPER activation ameliorates renal function in cisplatin-induced chronic kidney disease and suppresses renal fibrosis [11]. These findings underscore the pivotal role of GPER in modulating estrogen-responsive pathways, positioning it as a promising therapeutic target for pharmacological interventions. Some studies have shown that the renal protective effects of E2 are not mediated by ERα and ERβ [12]. Therefore, we hypothesize that the renal protective effects of E2 and RAL are mediated by GPER.

Studies have found that long-term treatment with E2 and RAL can reduce the severity of multi-organ dysfunction in ovariectomized rats with sepsis. This effect is attributed to the upregulation of heme oxygenase-1 (HO-1) protein, which exerts antioxidant and anti-inflammatory capabilities [13,14].

This study investigated whether the estrogen membrane receptor GPER pathway directly interacts with the nuclear factor erythroid 2-related factor 2 (Nrf2)/HO-1 pathway. To further clarify the role of estrogen, female mice were ovariectomized and then treated with E2 and RAL. Renal injury severity was compared in female mice following IRI, and the Nrf2/HO-1-related pathway was examined. Furthermore, the specific mechanism by which GPER regulates the Nrf2/HO-1 pathway was explored using GPER and Nrf2 inhibitors.

## 2. Results

### 2.1. E2 and RAL Pretreatment Upregulate the Expression of GPER in Renal Tissue

In this study, the ovariectomized (OVX) and IRI models were established (Supplementary Figure S1). Four weeks after OVX, IRI surgery was conducted to create an AKI model. After clamping the renal pedicles of the mice bilaterally, the color of the kidneys changed from red to dark purple; upon releasing the artery clamp, the kidneys turned red again, indicating successful IRI (Supplementary Figure S1). There were no significant differences in body weight among the groups on the first and last days of the experiment. There were also no significant differences in bilateral kidney weights, heart weights, and lung weights among the groups. However, the uterine weights showed statistically significant differences between groups. Compared to the Non OVX Sham group, the OVX Sham group, OVX IRI group, and RAL group showed reduced uterine and adnexal weights; in comparison to the OVX IRI group, the E2 group exhibited increased uterine and adnexal weights, with all differences being statistically significant (Table 1 and Supplementary Figure S1).

**Figure 1:**
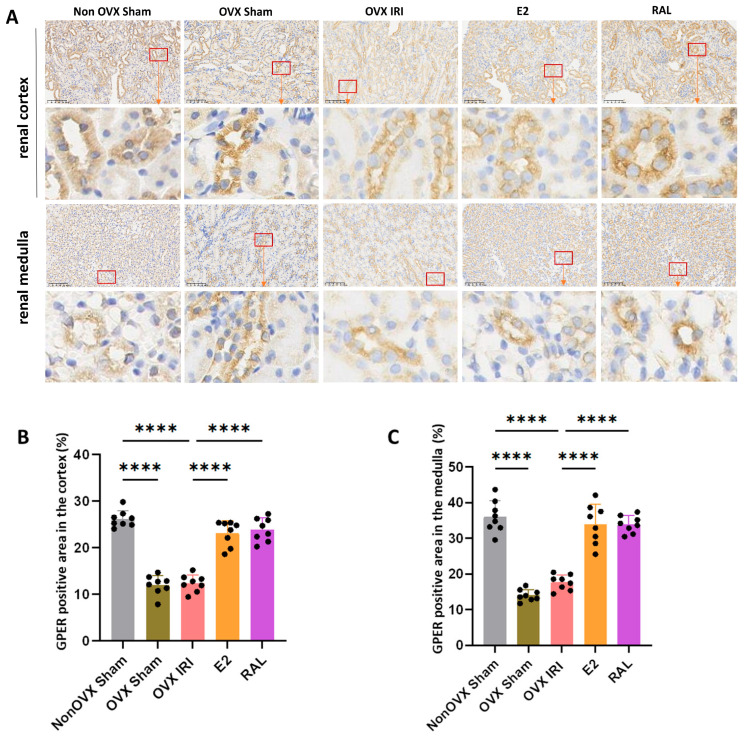
GPER Expression Patterns in the Renal Cortex and Medulla of Mice. (**A**) Immunohistochemistry results showing GPER expression in the renal cortex and medulla of mice. The red frame indicates renal tubules with positive GPER expression. GPER-positive staining was visualized as intense brown precipitate, which was specifically localized to the cytoplasmic and membranous compartments. Nuclei were counterstained blue with hematoxylin. (scale bar = 100 μm). (**B**) The GPER-positive area in the renal cortex of mice, n = 8. (**C**) The GPER-positive area in the renal medulla of mice, n = 8. Data were presented as $\bar{X}$ ± SD. Quantitative data were tested for normality and exhibited a normal distribution. One-way ANOVA was used to analyze differences among multiple groups, and pairwise comparisons were performed using Tukey’s HSD post hoc test. ‘****’: *p* < 0.0001.

Immunohistochemical results demonstrated positive GPER expression in both the renal cortex and medulla of the mice, characterized by deep cytoplasmic and membrane staining in tubular epithelial cells. The positive signal appeared as a deep brown color, predominantly localized to the cell membrane and cytoplasm of renal tubular epithelial cells (Figure 1A). Compared to the Non OVX Sham group, the percentage of GPER-positive stained area in the renal cortex and medulla was reduced in the OVX Sham group and OVX IRI group (*p* < 0.0001). This indicates that OVX surgery can lead to downregulation of GPER expression in the renal cortex and medulla of mice. Compared to the OVX IRI group, E2 and RAL pretreatments resulted in an increased percentage of GPER-positive stained area in the renal cortex and medulla of ovariectomized AKI mice (*p* < 0.0001) (Figure 1B,C).

### 2.2. ML385 and G15 Attenuate the Protective Effects of E2 and RAL Pretreatment on Renal Function and Renal Histology

**Figure 2:**
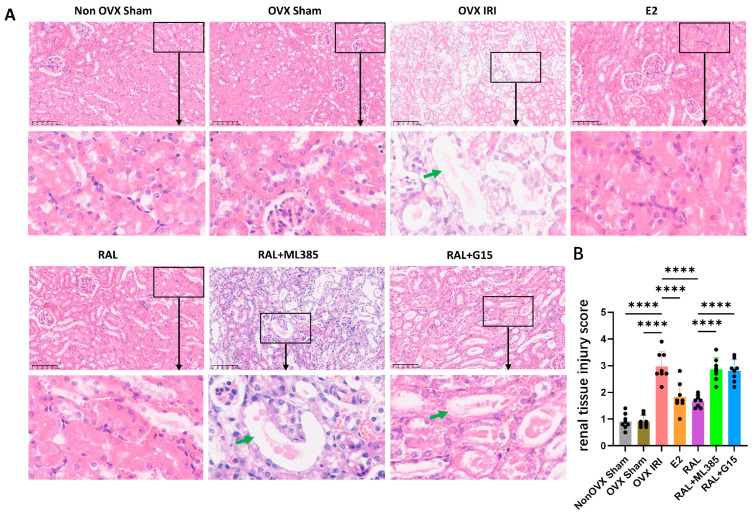
Analysis of kidney tissue HE staining in ovariectomized AKI mice. (**A**) HE staining of kidney tissue in mice. Non OVX Sham and OVX Sham groups showed intact, neatly arranged renal tubules with no dilation or protein casts. The OVX IRI group exhibited severe tubular injury, including dilation, epithelial necrosis, detachment, and massive protein casts (green arrows). E2 or RAL treatment attenuated these changes, preserving tubular structure and reducing casts. Co-treatment of RAL with ML385 or G15 led to the reappearance of severe tubular dilation, epithelial necrosis, and protein casts (green arrows). (scale bar = 100 μm). (**B**) Kidney injury scores, n = 8. Data were expressed as $\bar{X}$ ± SD. Quantitative data were tested for normality and exhibited a normal distribution. One-way ANOVA was used to analyze differences among multiple groups, and pairwise comparisons were performed using Tukey’s HSD post hoc test. ‘****’: *p* < 0.0001”.

The results of kidney HE staining show that the OVX IRI group, RAL + ML385 group, and RAL + G15 group had dilated renal tubules, thinning of the tubular epithelial cell cytoplasm, necrosis, and detachment, with large amounts of protein casts visible. These groups had higher kidney injury scores. In contrast, the Non OVX Sham group, OVX Sham group, E2 group, and RAL group showed neatly arranged renal tubules with no significant dilation, and fewer protein casts (Figure 2A). Compared with the Non OVX Sham group, the OVX IRI group exhibited a significantly higher renal tissue injury score (*p* < 0.0001). Similarly, the renal injury score was also markedly increased in the OVX IRI group relative to the OVX Sham group (*p* < 0.0001). In contrast, treatment with E2 or RAL notably reduced the renal injury score compared with the OVX IRI group (*p* < 0.0001). However, the protective effect of RAL was significantly reversed by ML385 or G15, as reflected by higher renal injury scores in the RAL + ML385 and RAL + G15 groups than in the RAL group (*p* < 0.0001) (Figure 2B).

**Figure 3:**
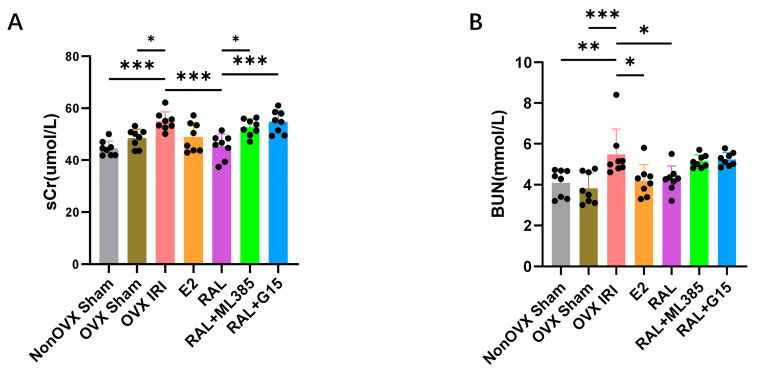
Analysis of renal function in ovariectomized AKI mice. (**A**) sCr concentrations in mice, n = 8. (**B**) BUN concentrations in mice, n = 8. Data were expressed as $\bar{X}$ ± SD. Quantitative data were tested for normality and exhibited a normal distribution. One-way ANOVA was used to analyze differences among multiple groups, and pairwise comparisons were performed using Tukey’s HSD post hoc test. ‘*’: *p* < 0.05, ‘**’: *p* < 0.01, ‘***’: *p* < 0.001.

Further analysis of renal function-related serum markers revealed that sCr (*p* < 0.001) and BUN (*p* < 0.01) levels were significantly increased in the OVX IRI group compared with the Non OVX Sham group. Compared with the OVX Sham group, sCr (*p* < 0.01) and BUN (*p* < 0.001) levels were also significantly elevated in the OVX IRI group. These sCr, BUN, and kidney pathology results indicate that the ovariectomized AKI mouse model was successfully established. Compared to the OVX IRI group, sCr (*p* < 0.001) and BUN (*p* < 0.05) levels were lower in the RAL group (Figure 3A,B). In contrast, sCr levels were higher in the RAL + ML385 group (*p* < 0.05) and RAL + G15 group (*p* < 0.001) compared to the RAL group (Figure 3A).

### 2.3. ML385 and G15 Attenuate the Anti-Inflammatory and Antioxidant Effects of E2 and RAL Pretreatment

**Figure 4:**
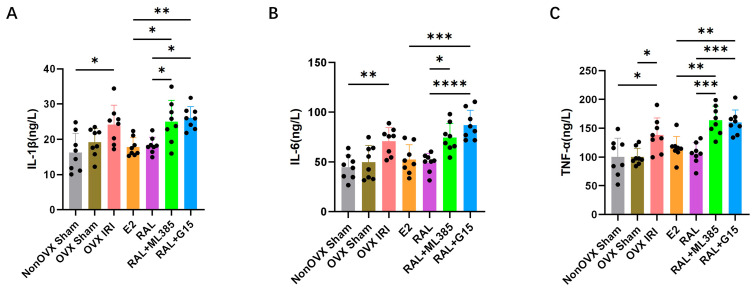
Analysis of inflammation responses in OVX-induced AKI mice. (**A**) Concentration of IL-1β in mouse serum, n = 8. (**B**) Concentration of IL-6 in mouse serum, n = 8. (**C**) Concentration of TNF-α in mouse serum, n = 8. Data are presented as $\bar{X}$ ± SD. Quantitative data were tested for normality and exhibited a normal distribution. One-way ANOVA was used to analyze differences among multiple groups, and pairwise comparisons were performed using Tukey’s HSD post hoc test. ‘*’: *p* < 0.05; ‘**’: *p* < 0.01; ‘***’: *p* < 0.001; ‘****’: *p* < 0.0001.

Serum inflammatory factor analysis showed that the levels of IL-1β (*p* < 0.05), IL-6 (*p* < 0.01), and TNF-α (*p* < 0.05) were significantly increased in the OVX IRI group compared with the Non OVX Sham group. Compared with the RAL group, serum levels of IL-1β (*p* < 0.05), IL-6 (*p* < 0.05), and TNF-α (*p* < 0.001) were all elevated in the RAL + ML385 group. Similarly, compared with the RAL group, serum levels of IL-1β (*p* < 0.05), IL-6 (*p* < 0.0001), and TNF-α (*p* < 0.001) were increased in the RAL + G15 group. Furthermore, compared with the E2 group, the RAL + G15 group exhibited elevated serum levels of IL-1β (*p* < 0.01), IL-6 (*p* < 0.001), and TNF-α (*p* < 0.01), whereas the RAL + ML385 group only showed increased serum levels of IL-1β (*p* < 0.05) and TNF-α (*p* < 0.01) (Figure 4A–C).

**Figure 5:**
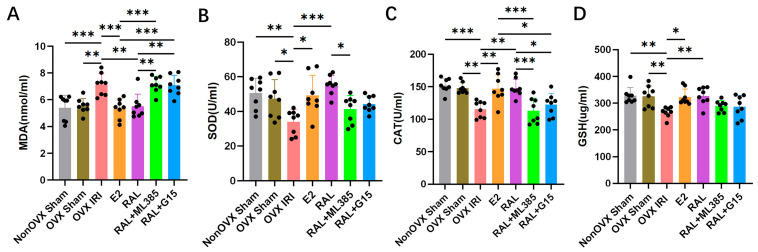
Analysis of oxidative stress responses in OVX-induced AKI mice. (**A**) Concentration of MDA in mouse kidney tissue, n = 8. (**B**) SOD activity in mouse kidney tissue, n = 8. (**C**) CAT activity in mouse kidney tissue, n = 8. (**D**) Concentration of GSH in mouse kidney tissue, n = 8. Data were presented as $\bar{X}$ ± SD. Quantitative data were tested for normality and exhibited a normal distribution. One-way ANOVA was used to analyze differences among multiple groups, and pairwise comparisons were performed using Tukey’s HSD post hoc test. ‘*’: *p* < 0.05; ‘**’: *p* < 0.01; ‘***’: *p* < 0.001.

The results for oxidative stress indicators in kidney tissue showed that, compared to the Non OVX Sham group, the OVX IRI group had elevated concentrations of MDA (*p* < 0.001), decreased SOD (*p* < 0.01) and CAT (*p* < 0.001) activity, and reduced GSH (*p* < 0.01) levels. Pretreatment with E2 and RAL exerted antioxidant effects in mice with OVX-induced AKI. In kidney tissue homogenates, compared with the OVX IRI group, the E2 group displayed reduced MDA content (*p* < 0.001), increased activities of SOD (*p* < 0.05) and CAT (*p* < 0.01), and elevated GSH levels (*p* < 0.05). Similarly, the RAL group exhibited decreased MDA content (*p* < 0.01), enhanced SOD (*p* < 0.001) and CAT (*p* < 0.01) activities, and higher GSH levels (*p* < 0.01). Compared to the RAL group, the levels of MDA in the serum of mice in the RAL + G15 group and the RAL + ML385 group were elevated (all *p* < 0.01), while the levels of SOD (*p* < 0.05) and CAT (*p* < 0.001) in the RAL + ML385 group were decreased, the level of CAT (*p* < 0.05) in the RAL + G15 group was decreased. Compared to the E2 group, the serum MDA levels in the RAL + ML385 group and the RAL + G15 group were elevated (all *p* < 0.001), and the CAT levels in the RAL + ML385 group (*p* < 0.001) and the RAL + G15 group (*p* < 0.05) were decreased (Figure 5A–D).

### 2.4. ML385 and G15 Attenuate the Protective Effects of E2 and RAL Pretreatment on the Ultrastructure of Renal Tissue

**Figure 6:**
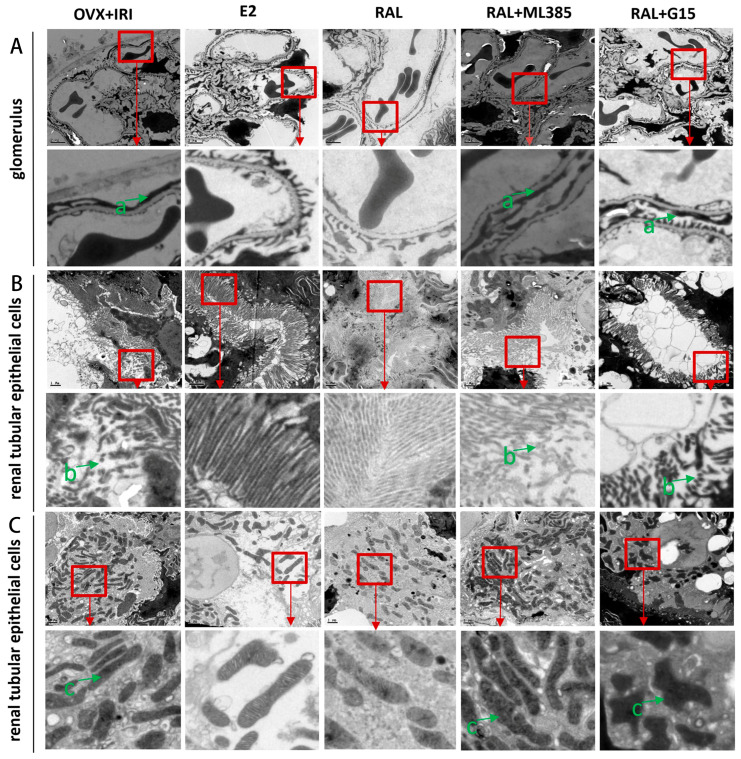
Electron Transmission Microscopy Analysis of Renal Tissue. (**A**) Glomerulus: The green arrow (a) indicates widespread fusion of podocyte foot processes (scale bar: 1 μm). (**B**) Renal Tubular Epithelial Cells: The green arrow (b) points to broken cilia in the renal tubular epithelial cells (scale bar: 1 μm). (**C**) Mitochondria in Renal Tubular Epithelial Cells: The green arrow (c) shows blurred cristae structures within the mitochondria of renal tubular epithelial cells (scale bar: 1 μm).

The study utilized electron transmission microscopy (TEM) to further examine the ultrastructural changes in the glomerular basement membrane and other renal regions. The results showed that in the E2 group and RAL group, the distribution of podocyte foot processes was more regular, whereas in the OVX IRI group, RAL + ML385 group, and RAL + G15 group, there was widespread fusion and disappearance of the podocyte foot processes (Figure 6A). Additionally, the morphology of proximal renal tubular epithelial cell cilia appeared intact in the E2 group and RAL group, while in the OVX IRI group, RAL + ML385 group, and RAL + G15 group, the cilia were fragmented and incomplete (Figure 6B). Lastly, in the E2 group and RAL group, the mitochondria of renal tubular epithelial cells were oval-shaped with clearly visible cristae. In contrast, in the OVX IRI group, RAL + ML385 group, and RAL + G15 group, the mitochondria appeared irregular, elongated, and antler-shaped, with blurred cristae structures (Figure 6C).

### 2.5. E2 and RAL Pretreatment Mediates and Activates the Nrf2/HO-1 Signaling Pathway Through GPER

**Figure 7:**
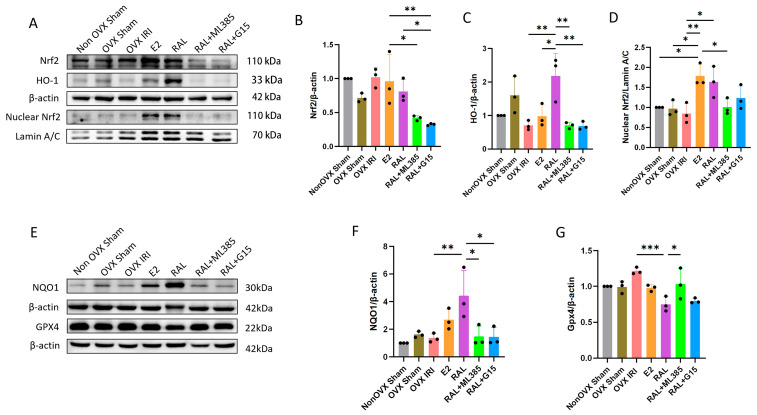
Effects of Nrf2, HO-1, NQO1, and Gpx4 Protein Expression in Renal Tissues of Ovariectomized AKI Mice. (**A**) Western Blot results showing the changes in Nrf2, Nuclear Nrf2, and HO-1 protein expression levels. (**B**) Quantitative analysis of Nrf2 expression using density measurement, n = 3. (**C**) Quantitative analysis of HO-1 expression using density measurement, n = 3. (**D**) Quantitative analysis of Nuclear Nrf2 expression using density measurement, n = 3. (**E**) Western Blot results showing the changes in NQO1 and Gpx4 protein expression levels. (**F**) Quantitative analysis of NQO1 expression using density measurement, n = 3. (**G**) Quantitative analysis of Gpx4 expression using density measurement, n = 3. Data were expressed as $\bar{X}$ ± SD. Quantitative data were tested for normality and exhibited a normal distribution. One-way analysis of variance (one-way ANOVA) was used to analyze differences among multiple groups, and pairwise comparisons were performed using Tukey’s HSD post hoc test. ‘*’: *p* < 0.05, ‘**’: *p* < 0.01, and ‘***’: *p* < 0.001.

The Western Blot results indicated that the nuclear Nrf2 protein levels were uniformly low in the Non OVX Sham group, OVX Sham group, and OVX IRI group. Following E2 intervention, nuclear Nrf2 protein content was significantly increased (*p* < 0.01), and RAL intervention also elevated nuclear Nrf2 protein expression (*p* < 0.05), demonstrating that both interventions effectively promote the nuclear translocation of Nrf2 to exert its transcriptional regulatory functions. In contrast, compared with the E2 group, nuclear Nrf2 protein expression was significantly decreased in the RAL + ML385 group (*p* < 0.05). These findings confirm that the stimulatory effect of RAL on Nrf2 nuclear translocation is dependent on both Nrf2 pathway activity and GPER mediation (Figure 7A,D).

Compared to the OVX IRI group, RAL pretreatment significantly increased the expression levels of HO-1 and NQO1 in renal tissue (all *p* < 0.01), while Gpx4 expression decreased (*p* < 0.001). Compared with the RAL group, the expression of Nrf2 (*p* < 0.05), HO-1 (*p* < 0.01), and NQO1 (*p* < 0.05) was reduced in the RAL + G15 group. In the RAL + ML385 group, the levels of HO-1 (*p* < 0.01) and NQO1 (*p* < 0.05) were decreased, while Gpx4 expression was increased (*p* < 0.05) (Figure 7A–G). These findings suggest that treatment with RAL in combination with ML385 or G15 can reverse the increase in HO-1 and NQO1 expression induced by RAL.

## 3. Discussion

AKI is one of the main complications in ICU patients, especially in critically ill individuals, and is associated with both short-term and long-term mortality. Therefore, researching the molecular mechanisms of AKI and developing preventive and therapeutic drugs is of great significance. After AKI occurs, the production of reactive oxygen species (ROS) leads to oxidative stress damage, causing cell necrosis, activation, and secretion of pro-inflammatory cytokines, which results in kidney tissue damage and dysfunction, further influencing the development and prognosis of kidney diseases.

GPER plays multiple roles in both estrogen-dependent and estrogen-independent physiological and pathological processes [15,16]. A deficiency in GPER can lead to various physiological changes, including obesity, cardiovascular dysfunction, insulin resistance, and impaired glucose tolerance [16,17]. In 2000, researchers discovered that estrogen binds to the orphan G-protein-coupled receptor GPR30, activating rapid cell signaling and initiating responses such as kinase activation and ion mobilization [18,19,20]. In 2008, the International Union of Basic and Clinical Pharmacology (IUPHAR) officially named this receptor GPER [21,22]. GPER is primarily expressed on the cell membrane, but is also found in the endoplasmic reticulum and Golgi apparatus.

GPER can be activated by various chemicals from different sources, including endogenous estrogens, phytoestrogens, mycoestrogens, and synthetic molecules with estrogenic activity. In this study, an endogenous estrogen-deficient model was created using OVX surgery, which resulted in the downregulation of GPER expression in the kidney tissue. However, after treatment with exogenous E2 and RAL, GPER expression in the kidney tissue was restored, indicating that GPER expression can be regulated by external E2 and RAL. Other steroids, such as estrone, estriol, testosterone, progesterone, aldosterone, and corticosterone, barely bind to GPER [23].

This study also found that pretreatment with exogenous E2 and RAL in mice with endogenous estrogen deficiency improved kidney tissue pathology and kidney function caused by AKI. The arrangement of podocyte foot processes in the glomeruli was more regular, with fewer signs of fusion. The structure of cilia in renal tubular cells remained relatively intact, and the mitochondrial cristae appeared more normal. Additionally, the release of inflammatory cytokines IL-1, IL-6, and TNF-α in mouse serum decreased, while MDA, a metabolic byproduct of oxidative stress, was reduced in kidney tissue. At the same time, the activities of antioxidant enzymes SOD and CAT increased, and GSH concentrations were elevated. However, after treatment with RAL combined with the GPER antagonist G15, the kidney-protective effects weakened or disappeared. This suggests that exogenous E2 and RAL not only regulate GPER expression but also enhance GPER activity, contributing to kidney protection in AKI.

GPER activation triggers several G proteins, leading to various downstream cascades, including the production of cyclic adenosine monophosphate (cAMP) and the activation of protein kinase A (PKA) and cAMP-response element binding protein (CREB). G protein activation also results in the mobilization of intracellular Ca^2+^, which activates protein kinase C (PKC) and leads to the activation of calcium channels. GPER can regulate renal artery and intrarenal vascular tension, increase the permeability of Ca^2+^ in renal tubular cells, and activate H^+^ATP enzyme activity [24]. The role of GPER in cellular signaling, molecular biology, pharmacology, and genetics has become a research focus. Recent studies suggest that GPER activation has renoprotective effects in various kidney injury models [25,26,27]. The role of GPER-specific agonists in pathophysiology and human disease could hold potential therapeutic value.

Under normal conditions, Nrf2 protein is unstable because Kelch-like ECH-associated protein 1 (Keap1) in the cytoplasm promotes the ubiquitination of Nrf2, preventing its translocation to the nucleus. Certain protein kinases, such as PKC, phosphorylate and activate Nrf2, protecting it from Keap1-mediated degradation [28]. Interventions with E2 or RAL effectively promote Nrf2 nuclear translocation to exert transcriptional regulatory functions by modulating downstream target genes. The stimulatory effect of RAL on Nrf2 nuclear translocation is dependent on both intrinsic Nrf2 pathway activity and specific GPER mediation. Combined intervention with RAL and the GPER antagonist G15 completely blocks the nuclear translocation and activation of Nrf2. These results are consistent with the notion that the stimulatory effect of E2 and RAL on Nrf2 nuclear translocation may involve GPER-mediated signaling.

This study found that after OVX surgery, Nrf2 expression decreases, likely due to reduced endogenous estrogen, which increases Nrf2 degradation. However, after IRI surgery, the inflammatory response reduces Nrf2 degradation. Similarly, exogenous E2 and RAL can also reduce Nrf2 degradation. The combined use of RAL and the GPER antagonist G15 effectively blocks Nrf2 activation in mouse kidney tissue, worsening kidney damage, increasing the release of inflammatory cytokines, and reducing antioxidant enzyme expression. This suggests that under ischemic and hypoxic conditions, GPER activation inhibits Nrf2 degradation in the kidney, thereby further activating a series of downstream antioxidant enzymes.

Nrf2 is considered the key regulator of the antioxidant response. Upon activation, Nrf2 translocates into the nucleus and binds to the antioxidant response element (ARE), leading to the upregulation of downstream antioxidant enzymes such as HO-1, Gpx4, SOD, CAT, and NQO1. These enzymes play a crucial role in defending against oxidative stress and contribute to the recovery from AKI [28,29]. Our study found that the combination of RAL and the Nrf2 inhibitor ML385 exacerbated kidney injury, as indicated by elevated sCr levels, increased levels of inflammatory cytokines, and reduced expression of antioxidant enzymes, such as HO-1 and NQO1, in both serum and kidney tissue.

Our study found that after OVX surgery, mice exhibited fat accumulation, which has been reported to induce low-grade chronic inflammation by enhancing the production of pro-inflammatory cytokines. This process disrupts the oxidant/antioxidant balance, leading to increased oxidative stress [30]. In addition to metabolic dysregulation, the inflammatory state and the tissue oxidant/antioxidant balance undergo dynamic changes in mice lacking endogenous estrogen. Early stages show compensatory increases in antioxidants, while later stages are marked by antioxidant depletion. Our results demonstrated that after OVX, Nrf2 expression decreased, whereas HO-1 and NQO1 were compensatorily upregulated. However, IRI surgery led to the depletion of antioxidants.

This study also found that after AKI in ovariectomized mice, Gpx4 expression in renal tissue increased, whereas Gpx4 expression decreased after E2 and RAL pretreatment, showing an opposite trend to HO-1 and NQO1. Notably, Gpx4 expression was restored following co-administration of ML385, consistent with the reversal of RAL’s renoprotective effects observed in our functional assays.

As a key antioxidant enzyme, Gpx4 plays a critical role in protecting cells and tissues from oxidative damage induced by free radicals, and its deficiency has been linked to multiple forms of cell death, including apoptosis, necroptosis, and ferroptosis, in preclinical models [31,32,33,34,35,36]. However, it is important to emphasize that no ferroptosis-specific markers or functional inhibition assays were included in the present study, which limits our ability to draw definitive conclusions regarding ferroptosis involvement in OVX-related renal IRI. Future studies incorporating ferroptosis-specific biomarkers and functional validation with ferroptosis inhibitors will be essential to verify whether the modulation of Gpx4 by E2 and RAL contributes to renal protection via ferroptosis regulation in OVX-related renal IRI.

In this study, the GPER antagonist G15 was used to investigate GPER’s role, as it can block GPER signaling without altering the genetic background, thereby providing direct evidence of its role in AKI. Furthermore, G15, a highly specific GPER antagonist, has been widely used in GPER-related research and can effectively elucidate GPER function.

The mouse model provided preliminary data for the study. In future research, we will extend to rat models and further validate the specific mechanism by which E2 and RAL mediate the Nrf2/HO-1 pathway via GPER in in vitro models such as renal tubular epithelial cells and glomerular cells.

This study did not investigate the long-term side effects of the therapeutic regimen. However, the literature evidence suggests that long-term use of estrogens and their receptor modulators may be associated with risks such as malignancies and thrombosis. In future experimental designs, we will incorporate these factors and comprehensively evaluate the safety through long-term animal experiments and clinical follow-up studies.

Ideal hormone therapy should maintain the beneficial effects of estrogen (such as alleviating menopausal symptoms and preventing osteoporosis) without causing hyperplasia of the uterine and breast epithelial cells. This study found that E2 treatment led to endometrial hyperplasia and increased uterine size, whereas RAL treatment did not. Previous studies suggest that this balance can be achieved through tissue-selective estrogen complexes (TSECs), which combine SERMs with one or more estrogens [37]. SERMs are considered a way to prevent estrogen-induced carcinogenesis, and the TSEC approach is believed to offer estrogen’s protective effects on vital organs, like the kidneys, while preventing cancer and endometrial hyperplasia.

The enhancement of Nrf2 nuclear translocation capacity is a key link in the renoprotective effects of E2 and RAL. This discovery offers a novel target direction for the clinical development of preventive and therapeutic drugs for AKI. Clinically, AKI patients are often accompanied by excessive activation of oxidative stress. Although E2, as an endogenous estrogen, may cause side effects such as endometrial hyperplasia when used in hormone replacement therapy, RAL, as a selective estrogen receptor modulator, does not induce abnormal uterine tissue proliferation while promoting Nrf2 nuclear translocation and enhancing renal antioxidant capacity, demonstrating superior clinical application potential. We emphasize the experimental nature of this study and the need to confirm its efficacy in humans.

## 4. Materials and Methods

### 4.1. Animal Models and Grouping

Fifty-six adult female C57BL/6 mice (6–7 weeks, 16–18 g) were purchased from Beijing Vital River Laboratory Animal Technology Co., Ltd. (Beijing, China) and housed under specific pathogen-free conditions at the First Affiliated Hospital of Wenzhou Medical University. Mice were maintained at 25 °C, 55% humidity, and a 12-h light/dark cycle with ad libitum chow and water. Four to five mice were assigned per cage, with bedding changed twice weekly. Food was withheld for 12 h before surgery.

After one week of acclimatization, mice were anesthetized with 0.3% pentobarbital sodium (50 mg/kg, intraperitoneally). Bilateral OVX was performed, except in the Non OVX Sham group, where ovaries were exposed but not removed. Four weeks later, IRI was induced by clamping the renal pedicles for 45 min [38,39]. Ischemia and reperfusion were confirmed by color changes. In sham-operated mice, renal pedicles were isolated without occlusion.

**Figure 8:**
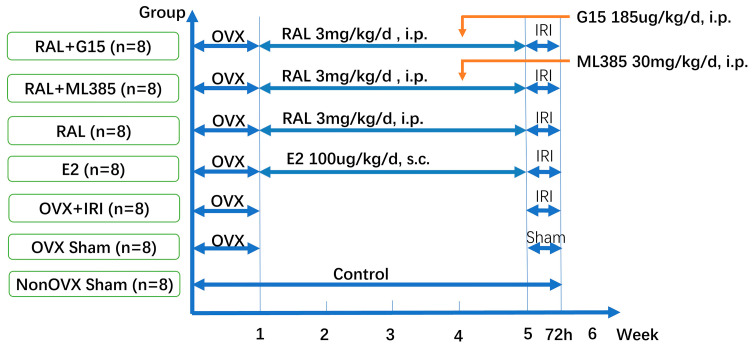
Experimental protocol of the study.

Mice were randomly divided into seven groups (n = 8): Non OVX Sham, OVX Sham, OVX IRI, E2 (OVX + IRI + 17β-estradiol), RAL (OVX + IRI + raloxifene), RAL + ML385 (OVX + IRI + raloxifene + ML385), and RAL + G15 (OVX + IRI + raloxifene + G15). Group allocation was performed using a random number table. One week after OVX, pharmacological interventions were started. E2 group received 17β-estradiol (Sigma, St. Louis, MO, USA) 100 μg/kg/day, subcutaneously for 4 weeks. RAL group received raloxifene (GLPBIO, Montclair, CA, USA) 3 mg/kg/day, intraperitoneally for 4 weeks. RAL + ML385 and RAL + G15 groups received raloxifene for 4 weeks, with ML385 (GLPBIO, Montclair, CA, USA) 30 mg/kg/day or G15 (MCE, Monmouth Junction, NJ, USA) 185 μg/kg/day in the final week. After pretreatment, mice underwent bilateral renal IRI or sham surgery (Figure 8). Subcutaneous injection of buprenorphine (Sigma, St. Louis, MO, USA) 0.05 mg/kg was administered every 12 h for 2 consecutive days following the surgery.

At 72 h post-IRI, mice were euthanized by intraperitoneal injection of 0.3% pentobarbital sodium (200 mg/kg). At the end of the experiment, after the mice were euthanized, they were promptly dissected. Bilateral kidneys, hearts, uteri, and lungs were completely isolated and accurately weighed using an electronic balance. All procedures were approved by the Animal Ethics Committee of the First Affiliated Hospital of Wenzhou Medical University (Approval No. WYYY-IACUC-AEC-2024-031).

### 4.2. Renal Histopathology

One kidney was immediately immersed in 4% paraformaldehyde fixative solution and fixed at room temperature for 12–24 h. The study employed a blinded scoring method, using an optical microscope at ×200 magnification to observe kidney tissue sections. Renal tissue damage was characterized by the loss of brush borders, tubular dilation, necrosis, and shedding of tubular epithelial cells, tubular cast formation, and vacuolization. Researchers, unaware of the group assignments, randomly selected 10 fields of view from the HE-stained sections of each mouse and scored kidney damage based on the percentage of damaged tubule area in the renal cortical tubular epithelial cells, calculating the average score as follows: 0 points (normal kidney); 1 point (damaged tubular epithelial cell area < 25%); 2 points (damaged tubular epithelial cell area 25–50%); 3 points (damaged tubular epithelial cell area 50–75%); 4 points (damaged tubular epithelial cell area > 75%).

### 4.3. Western Blot Analysis

From the contralateral unfixed kidney, fresh kidney tissue fragments were immediately aliquoted and stored at −80 °C for Western Blot analysis. Fresh kidney tissue fragments were immediately transferred into cryopreservation tubes containing precooled lysis buffer. Total protein was extracted using RIPA buffer containing protease inhibitors (Beyotime, Beijing, China). Protein concentration was measured with a BCA assay kit (Beyotime). Equal amounts of protein (30 μg) were separated by 10% SDS-PAGE and transferred to PVDF membranes. Membranes were blocked with non-fat milk, cut according to molecular weight, and incubated overnight at 4 °C with primary antibodies against Nrf2, HO-1, NQO1, and Gpx4 (all 1:1000, Abcam, Cambridge, UK). After washing, membranes were incubated with HRP-conjugated secondary antibodies (goat anti-mouse 1:3000 or goat anti-rabbit 1:5000). Band intensities were quantified using ImageJ software, version 1.54f and normalized to β-actin. The data were representative of three independent experiments.

### 4.4. Transmission Electron Microscopy (TEM) Analysis

From the contralateral unfixed kidney, small fresh tissue fragments were cut into 1 mm^3^ pieces using a sterile scalpel within 1–3 min, then rapidly immersed in precooled 2.5% glutaraldehyde fixative at 4 °C. The tissue fragments were dehydrated in a graded ethanol-acetone series, embedded in resin, and sectioned with an ultramicrotome. Kidney cortical samples (2 mm × 2 mm) were harvested within 1–3 min, fixed in 2.5% glutaraldehyde at 4 °C, dehydrated through graded ethanol and acetone, embedded in resin, and sectioned using an ultramicrotome. Ultrathin sections were mounted on metal grids and examined by transmission electron microscopy (JEOL, Tokyo, Japan) at the Electron Microscopy Core Facility of Wenzhou Medical University.

### 4.5. Immunohistochemistry

The IHC examination was performed on kidney tissue fragments fixed in formaldehyde. Kidney sections were deparaffinized, rehydrated, and subjected to antigen retrieval and protein blocking. Sections were incubated overnight at 4 °C with anti-GPER antibody (1:500, Bioss, Beijing, China), followed by secondary antibody incubation and chromogenic detection. Immunohistochemical sections were quantitatively analyzed using ImageJ. Ten non-overlapping fields were randomly selected from each section. The grayscale threshold was uniformly set, and the percentage of GPER-positive stained area relative to the total tubular area was calculated. The average value was used as the final positive expression rate for each sample.

### 4.6. Detection of sCr and BUN

After the disappearance of the corneal reflex, the chest skin was disinfected, and a syringe was inserted into the heart through the intercostal space to slowly aspirate blood. Serum Cr and BUN levels were measured using commercial kits (Muke, Wenzhou, China) and an automated biochemical analyzer (Siemens, Munich, Germany). The control group values were taken as a benchmark for statistical analysis.

### 4.7. Enzyme-Linked Immunosorbent Assay (ELISA)

Serum IL-1β, IL-6, and TNF-α concentrations were determined by enzyme-linked immunosorbent assay (ELISA) kits (Beyotime, Shanghai, China) according to the manufacturer’s instructions. Absorbance was measured at 450 nm using a microplate reader (Thermo, Waltham, MA, USA). Statistical analysis was based on control group values.

### 4.8. Oxidative Stress Assessment

The kidney sampling method was the same as that for the Western Blot assay. Renal MDA, SOD, CAT, and GSH levels were measured using commercial kits (Beyotime, Shanghai, China) following the manufacturers’ protocols. MDA was assessed by the Thiobarbituric acid (TBA) method (535 nm), SOD by the xanthine oxidase method (550 nm), CAT by the ammonium molybdate method (405 nm), and GSH by the modified Beutler method (412 nm). The control group served as the reference for statistical comparison.

### 4.9. Statistical Analysis

Data were analyzed using GraphPad Prism 10.0 and are presented as mean ± SD. Quantitative data were tested for normality. One-way ANOVA was used to analyze differences among multiple groups, and pairwise comparisons were performed using Tukey’s HSD post hoc test. Significant differences are indicated as ‘*’: *p* < 0.05, ‘**’: *p* < 0.01, ‘***’: *p* < 0.001, and ‘****’: *p* < 0.0001.

## 5. Conclusions

This study explored the renoprotective effects of E2 and RAL on OVX AKI mice through GPER-mediated activation of the Nrf2/HO-1 signaling pathway. Endogenous E2 deficiency reduces renal GPER expression, while E2 and RAL treatments restore it. Both agents exert renoprotective effects by reducing inflammation, improving renal function and histology, and enhancing antioxidant defense. Mechanistically, E2 and RAL promote Nrf2 nuclear translocation to activate downstream antioxidant genes, suggesting involvement of GPER signaling and Nrf2 pathway activity. These observations support a potential role of the GPER-Nrf2-HO-1 axis in protecting against OVX-related renal IRI. Further validation of these findings in human studies is required.

## Figures and Tables

**Table 1:** Body Weights and Major Organ Weights of Mice in Each Group After E2 and RAL Pretreatment.

Groups	Non OVX Sham (n = 8)	OVX Sham (n = 8)	OVX IRI (n = 8)	E2 (n = 8)	RAL (n = 8)	*p* Value
Body weight (g)	19.3 ± 1.3	20.5 ± 1.2	19.5 ± 1.4	19.8 ± 2.2	19.2 ± 2.1	0.575
Kidneys weight (g)	0.28 ± 0.04	0.25 ± 0.02	0.29 ± 0.05	0.30 ± 0.05	0.25 ± 0.03	0.056
Uterus weight (g)	0.13 ± 0.06	0.04 ± 0.03 *	0.08 ± 0.06 *	0.16 ± 0.06 ^@^	0.05 ± 0.02 *	0.000
Heart weight (g)	0.15 ± 0.02	0.15 ± 0.02	0.14 ± 0.02	0.14 ± 0.02	0.14 ± 0.01	0.309
Lungs weight (g)	0.11 ± 0.03	0.10 ± 0.02	0.10 ± 0.03	0.09 ± 0.03	0.11 ± 0.02	0.865

Data were presented as $\bar{X}$ ± SD. Quantitative data were tested for normality and exhibited a normal distribution. One-way analysis of variance (one-way ANOVA) was used to analyze differences among multiple groups. ‘*’ indicates a statistically significant difference compared to the Non OVX Sham group; ‘@’ indicates a statistically significant difference compared to the OVX IRI group.

## Data Availability

The original contributions presented in this study are included in the article. Further inquiries can be directed to the corresponding authors.

## Supplementary Materials

The following supporting information can be downloaded at https://www.mdpi.com/article/10.3390/ijms27073070/s1.

## Abbreviations

The following abbreviations are used in this manuscript:

Nrf2	Nuclear Factor Erythroid 2-Related Factor 2
HO-1	Heme Oxygenase-1 Activation
GPER	G Protein-Coupled Estrogen Receptor
IRI	Renal Ischemia–Reperfusion Injury
AKI	Acute Kidney Injury
RAL	Raloxifene
E2	Estradiol
OVX	ovariectomized
IL-1β	Interleukin-1β
IL-6	Interleukin-6
TNF-α	Tumor necrosis factor-alpha
MDA	Malondialdehyde
SOD	Superoxide dismutase
CAT	Catalase
GSH	Glutathione
NQO1	NAD(P)H: quinone oxidoreductase 1
TEM	Transmission Electron Microscopy
ELISA	Enzyme-Linked Immunosorbent Assay
TBA	Thiobarbituric acid
ANOVA	Analysis of Variance
