# Supplementary figures and images for: Estradiol and Raloxifene Protect Ovariectomized Mice from Acute Kidney Injury via G Protein-Coupled Estrogen Receptor-Mediated Nuclear Factor Erythroid 2-Related Factor 2/Heme Oxygenase-1 Activation
Source: Int J Mol Sci. 2026 Mar 27;27(7):3070. doi: 10.3390/ijms27073070 (PMC13073082; doi:10.3390/ijms27073070)

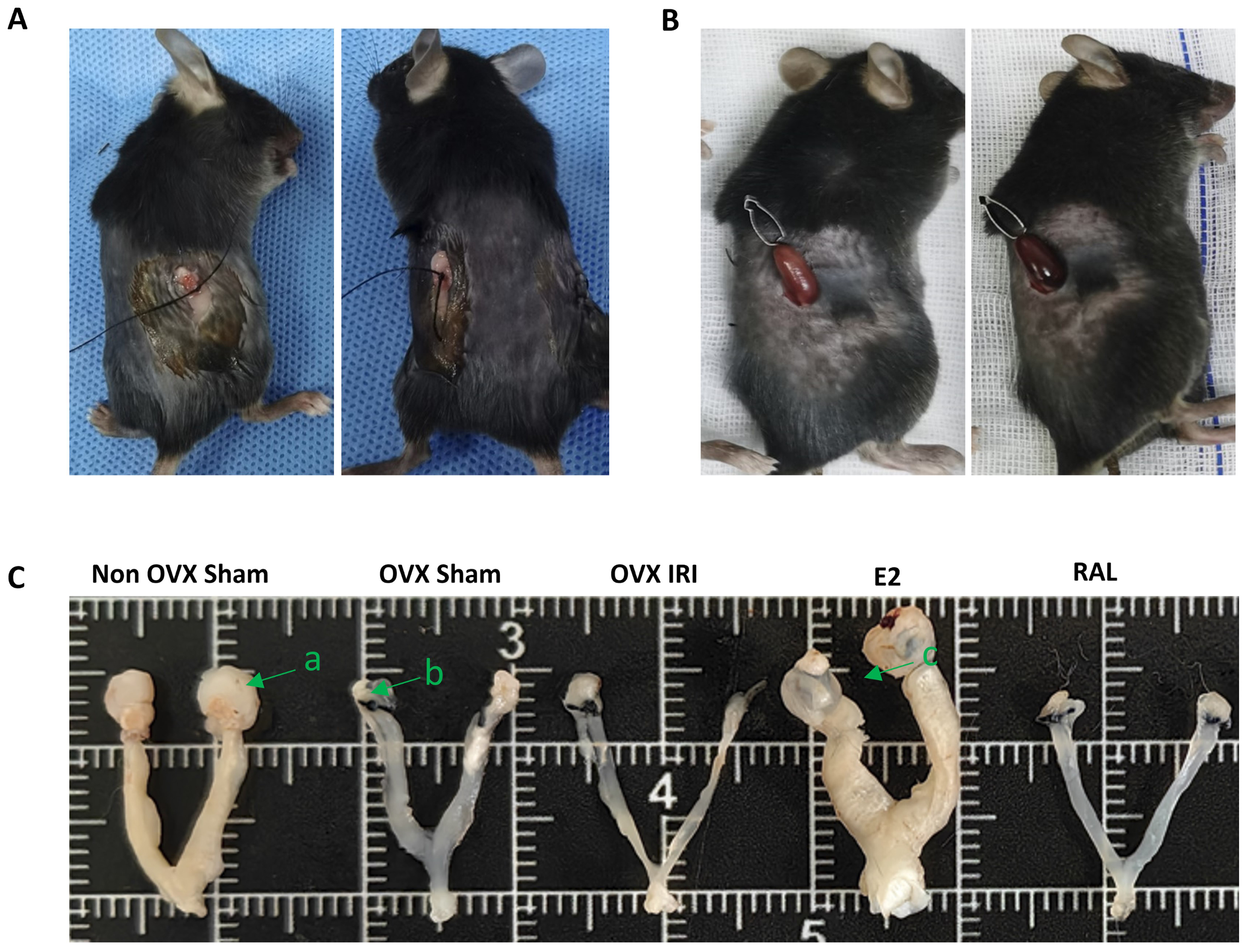

Supplement: Supplementary file 1 [file ijms-27-03070-s001.zip › ijms-4141902-supplementary.jpg]
